# The bridge symptoms of childhood trauma, sleep disorder and depressive symptoms: a network analysis

**DOI:** 10.1186/s13034-023-00635-6

**Published:** 2023-07-04

**Authors:** Weilong Guo, Yixin Zhao, Hui Chen, Jiali Liu, Xianliang Chen, Huajia Tang, Jiansong Zhou, Xiaoping Wang

**Affiliations:** Department of Psychiatry, National Clinical Research Center for Mental Disorders, The Second Xiangya Hospital of Central South University, Changsha, 410011 Hunan China

**Keywords:** Childhood trauma, Sleep disorder, Adolescent, Symptom network, Bridge symptoms

## Abstract

**Background:**

This study aimed to elucidate the characteristics of symptom network of childhood trauma (CT) and sleep disorder (SD) in Chinese adolescents, with the influence of depressive symptoms taken into account.

**Method:**

A total of 1301 adolescent students were included, and their CT, SD and depressive symptoms were measured using the Pittsburgh sleep quality index (PSQI), the Childhood Trauma Questionnaire-Short Form (CTQ-SF), and The Patient Health Questionnaire-9 (PHQ-9), respectively. Central symptoms and bridge symptoms were identified based on centrality indices and bridge centrality indices, respectively. Network stability was examined using the case-dropping procedure.

**Results:**

In CT and SD symptom network, emotional abuse and sleep quality symptoms had the highest centrality values, and two bridge symptoms, i.e., emotional abuse and sleep disturbance symptoms, were also identified. In symptom network for CT, SD, and depressive symptoms, sleeping difficulty symptoms, daily dysfunction symptoms, and emotional abuse appeared to be potential bridge symptoms. In symptom network of CT, SD, and depressive symptoms (excluding the symptom of sleeping difficulty), daily dysfunction symptoms, emotional abuse, and sleep disturbance symptoms appeared to be bridge symptoms.

**Conclusions:**

In this study, emotional abuse and poor sleep quality were found to be central symptoms in the CT-SD network structure among Chinese adolescent students, with daytime dysfunction as the bridge symptom in the CT-SD-depression network structure. Systemic multi-level interventions targeting the central symptoms and bridge symptoms may be effective in alleviating the co-occurrence of CT, SD and depression in this population.

**Supplementary Information:**

The online version contains supplementary material available at 10.1186/s13034-023-00635-6.

## Introduction

Childhood trauma(CT) is a global concern; a large number of cross-sectional survey studies found that one-third to one-half of the studied population had experienced CT [1–4], and the same results were also found in many cohort studies [5–10]. Studies have found that CT could impact the physical and mental health of people throughout their life [8, 9]. Sleep disorders, including difficulty falling asleep, low sleep efficiency, and daytime dysfunction, are found after the exposure to CT, and this can happen in a short term or in the long run [11]. Thus, sleep disorder (SD) may also be a pathway from CT to severe clinical symptoms or disease states and functional impairment [12].

Previous studies have found a strong link between CT and poor sleep quality, especially in adults. For example, Boynton-Jarrett et al. found that childhood sexual abuse was related to interrupted sleep in adulthood [13], and Rachel et al. reported that a majority of studies (88%) indicated a significant association between childhood sexual abuse and a variety of sleep disturbances (such as difficulty falling asleep, poor sleep quality, and nightmares) in a systematic review [14]. However, there have been relatively few studies on adolescents to date. Darlynn et al. found that CT appeared to be related to multiple aspects of sleep in adolescence [15], Susan et al. found the association of specific and cumulative CT with insufficient sleep duration using a nationally representative sample of American youth [16], and Park et al. reported that the relationship between CT and sleep problems could be partially mediated by depression and anxiety [17]. Although these studies have found that CT could affect the sleep pattern in adolescents, the sources of samples, sample sizes, and methodologies are different, leading to a lack of strong evidence on adolescents. Furthermore, most of prior studies focused on sleep duration and insomnia symptoms (e.g., difficulty falling asleep or maintaining sleep), and few have looked at sleep dimensions such as sleep efficiency, daytime dysfunction, and sleep medication.

Considering the impact of CT, the exploration of CT, SD and depression using a group of adolescents who have experienced or are experiencing CT might be more helpful, and more suitable interventions can be identified for this group through the in-depth exploration of the impact of different forms of childhood abuse on different sleep dimensions of adolescents. Meanwhile, the heterogeneity of trauma as well as its impacts might have led to great differences in the degree of damage to the mental health of individuals [18]; therefore, we look to explore the network structure between CT and SD using the approach of network analysis as well as to identify the bridge of the symptoms, in the hope of developing effective treatment strategies.

Network analysis has attracted great attention and been applied more frequently as a novel approach to understanding the nature and treatment in the spiritual psychology field over the past decade [19]. Network analysis has provided new insights into the functional role and importance of specific symptoms in maintaining diseases (e.g., the centrality of symptoms in the network) [20, 21]. For example, Kathryn et al. found that symptoms related to body shape and weight concerns as well as guilt are central eating disorder (ED) symptoms, while physical symptoms, self-esteem, and emotional overwhelm might underlie the comorbidities of EDs [22]. Courtney et al. found that anhedonia, low mood, and overwhelming feelings of worry were the most central symptoms of depression and anxiety [23]. Hyu Jung Huh et al. found that childhood emotional trauma might play an important role in the development of severe depressive symptoms [24].

Thus, in the present study, we aim to (a) identify the core symptoms of CT and SD in Chinese adolescents, (b) explore the direct bridge symptoms between CT and SD, and (c) identify the direct bridging symptoms of CT and SD with the impact of emotional state taken into account.

## Methods

### Subjects

The present study adopted a cross-sectional design. All the participants were recruited from a middle school in Changsha, China, from August 2021 to October 2021, and data were collected using cluster sampling. The Chinese online platform “Wenjuanxing” was used to collect information and responses from all participants, and the link was sent to the parents of the adolescents. Before the adolescent students started the questionnaire accompanied by their parents, the parents were required to read a brief introduction of the study and click the “Agree” button. All participants completed the relevant questionnaire independently, and their parents only accompanied them without intervening or commenting on their choices. The adolescents could be included in this study if they met the following criteria: [1] aged between 12 and 16 years, [2] in Grades 7 to 9, and [3] able to fully understand the content of the questionnaire. Adolescents meeting any of the following criteria were excluded: [1] refusing to participate in the survey, [2] failing to complete the questionnaire carefully or providing responses with poor quality. This study was part of the China Depression Cohort Study (CDCS) and was approved by the ethics committee of The Second Xiangya Hospital of Central South University.

### Methods

#### Sociodemographic data

The sociodemographic data were collected using a self-designed questionnaire, which included age, gender, grade, and history of smoking and drinking.

### Clinical data

Clinical data, including sleep parameters, experience of childhood trauma, and depression, were assessed using the following scales.

#### Pittsburgh sleep quality index (PSQI)

The PSQI scale was developed by Buysse et al. of the University of Pittsburgh in 1989 [25], and is mainly used to measure the sleep quality in the last month for people aged over 6 years [26]. It consists of 24 items, including 19 self-evaluated items and 5 items evaluated by others; 18 of the 19 self-evaluated items cover 7 dimensions, namely sleep quality (SQ), sleep latency (SL), sleep duration (SDU), sleep efficiency (SE), sleep disturbance (SDI), sleep medication (SM), and daily dysfunction (DD). The score of each dimension ranged from 0 to 3, and thus the total score ranged from 0 to 21; in this scale, higher scores indicated poorer sleep quality. For the validated Chinese version used in the present study, a cutoff score of 7 was used to determine whether a participant had SD symptoms [27]. The Cronbach’s alpha for this scale is 0.758.

#### The Childhood Trauma Questionnaire - Short Form (CTQ-SF)

The CTQ-SF was developed by David et al. of Fordham University in 2003, and is mainly used to measure the history of childhood trauma in people aged over 12 years [28]. It consists of 28 items, with 25 of them used to measure child abuse (overall); the 25 items are divided into 5 subscales assessing emotional abuse (EA), physical abuse (PA), sexual abuse (SA), emotional neglect (EN) and physical neglect (PN), with 5 items in each subscale. The score of each item ranged from 1 (‘never’) to 5 (‘very often’), and the score of each subscale was the sum of the scores of all 5 items in this subscale, with the scores of seven items (Items 2, 5, 7, 13, 19, 26 and 28) reversed; thus, the total score of each subscales ranged between 5 and 25. For validated Chinese version used in this study, the cutoff scores were 8 for PA, 8 for PN, 8 for SA, 10 for EA, and 15 for EN, to determine whether a participant was affected by the corresponding abuse and neglect [29]. The Cronbach’s alpha for this scale is 0.81.

#### The Patient Health Questionnaire-9 (PHQ-9)

The PHQ-9 was developed by Kroenke et al. of Indiana University in 2001[30], and is mainly used as a screening tool for depression for people aged over 12 years [31]. It consists of 9 self-evaluated items covering 9 depression modules, namely low interest or pleasure, feeling down and hopeless, sleeping difficulty, lack of energy, poor appetite/overeating, guilt, difficulty in concentrating, moving slowly/restlessness, and suicidal thoughts. Each item in these scales was rated on a 4-point Likert scale, with a score ranging from 0 ‘not at all’) to 3 (‘nearly every day’) and higher scores indicating higher severity of symptoms. For the validated Chinese version used in this study, a cutoff score of 5 was used to determine whether a participant had depressive symptoms [32]. The Cronbach’s alpha was 0.915.

A total of 1313 middle school students’ data was collected in this study. After excluding 12 erroneous data, a total of 1301 middle school students’ PSQI, CTQ, and PHQ-9 data were included in this study [33].

### Statistical analyses

To ensure the quality of our survey, all the completed questionnaires were screened for inappropriate responses and lack of response variation to open-ended questions. The descriptive analysis of demographic information and statistical analysis of scores of scales were performed using SPSS version 26.0 software.

### Network estimation

Network models were constructed and analyzed using the qgraph package(version 1.9.2) in the R software (version 4.2.2) [34, 35].

The partial correlation network method was used to estimate all symptom network, with the edges in the network representing associations between symptoms after adjustment for all other links. For the estimation of each partial correlation network, Gaussian graph models (GGMS) were first used to estimate the pairwise correlation parameters between nodes. As parameter estimation of all edges could lead to type I error, we used a graphical lasso [35, 36] to create a more parsimonious network by reducing weak correlations to exactly zero. The Fruchterman-Reingold algorithm was used to create the graphs [37].

In this network, a circle represented an individual symptom (one item from the symptom measures) from the PSQI, CTQ-SF and PHQ-9. The associations between nodes were represented by lines (or “edges”) between nodes. The edges represent dependencies between variables; blue edges indicate positive associations, and red edges indicate negative associations. Wider edges indicated stronger associations. Abbreviations were used to designate each of the 7 PSQI dimensions, 5 CTQ-SF dimensions and 9 PHQ-9 items in the study. These abbreviations are used in figures depicting the centrality values of nodes.

### Network centrality estimation

We employed three commonly used centrality measures, i.e., strength, closeness, and betweenness, to quantify the features of the nodes [38]. “Strength” represents the total weights of connections from other nodes to a specific node. “Closeness” is defined as the inverse of the sum of the shortest distances from a particular node to all other nodes in the network, where the shortest distance is the minimal number of edges traversed from one node to the next. “Betweenness” refers to the number of times that the shortest path between any two symptoms passes through another symptom.

### Network accuracy and stability estimation

The accuracy of edges and stability estimates for the network were calculated using the bootnet R package (version 1.5)[39] with 1000 iterations. The accuracy of the edges was examined using the 95% confidence interval (CI) of the bootstrap edge weight, with a narrower edge weight CI indicating higher accuracy. Then, we tested the stability of the centrality using correlations between centrality indices for the whole sample and indices for networks with a decreasing number of cases, with higher correlations between original indices and indices obtained from down sampling suggesting higher proneness to stable. The centrality stability coefficient (CS-coefficient) was also calculated as a reference index. A CS-coefficient [40] below 0.25 indicated high instability, and a value greater than or equal to 0.5 is recommended.

## Results

The demographic characteristics of all participants are shown in Table 1. The mean score of each dimension in the PSQI and CTQ-SF are shown in Table S1, and the mean score of each symptom in the PHQ-9 are shown in Table S2. Overall, 55.7% of the adolescents had experienced CT, 27.7% of them had SD, and 13.3% of them reported both adverse experiences.

**Table 1:** Demographic characteristics (n = 1301)

Variables	M (SD) or N (%)
Age (years)	13.36 (0.71)
Gender	
Female	696 (53.5)
male	605 (46.5)
Education (years)	7.90 (0.31)
Smoking	
Smoker	27 (2.1)
Non-smoker	1274 (97.9)
Drinking	
Drinker	42 (3.2)
Non-drinker	1259 (96.8)
CTQ-SF	725 (55.7)
PSQI	329 (27.7)
PHQ-9	249 (19.1)
GAD-7	287 (22.1)

### Model 1. Psychopathology of sleep disorder and childhood trauma

**Fig. 1:**
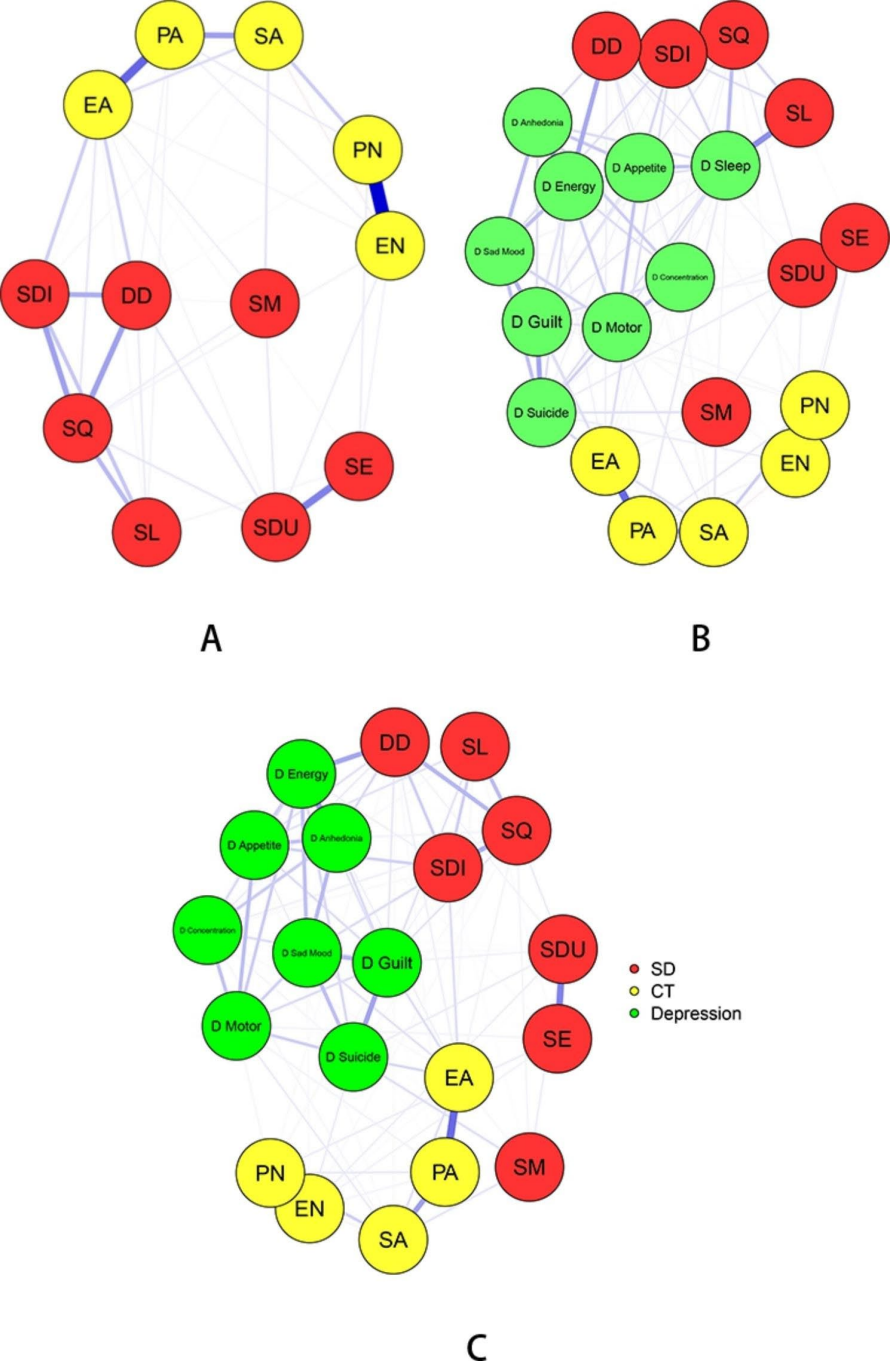
The sleep and abuse network, the sleep, abuse and depression Network (1), the sleep, abuse and depression network (2). Note. Labels for sleep symptoms: SQ = sleep quality, SL = sleep latency, SDU = sleep duration, SE = sleep efficiency, SDI = sleep disturbance; SM = sleep medication; DD = daytime dysfunction. Labels for abuse: EA = emotional abuse, PA = physical abuse, SA = sexual abuse, EN = emotional neglect, PN = physical neglect. Strength is indicated by the thickness of lines between nodes, with thicker lines representing stronger ties. Labels for depressive symptoms: D Anhedonia = low interest or pleasure; D Sad Mood = feeling down and hopeless; D Sleep = sleeping difficulty; D Energy = lack of energy; D Appetite = poor appetite/overeating; D Guilt = guilt; D Concentration = difficulty in concentrating; D Motor = moving slowly/restlessness; D Suicide = suicidal thoughts. Strength is indicated by the thickness of lines between nodes, with thicker lines representing stronger ties

**Fig. 2:**
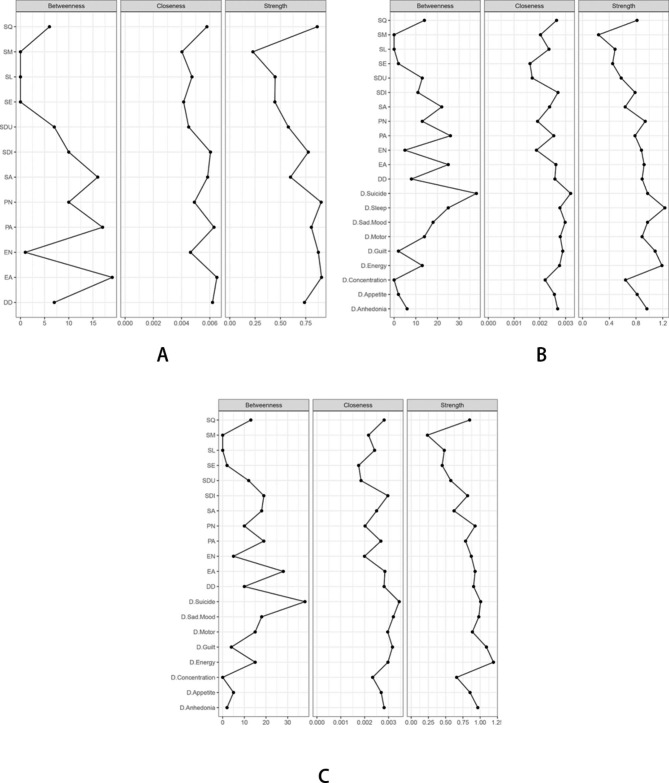
Centrality indices of the sleep and abuse network, sleep, abuse and depression network (1), sleep, abuse and depression network (2). Note: Values shown on the x-axis are standardized z-scores

**Fig. 3:**
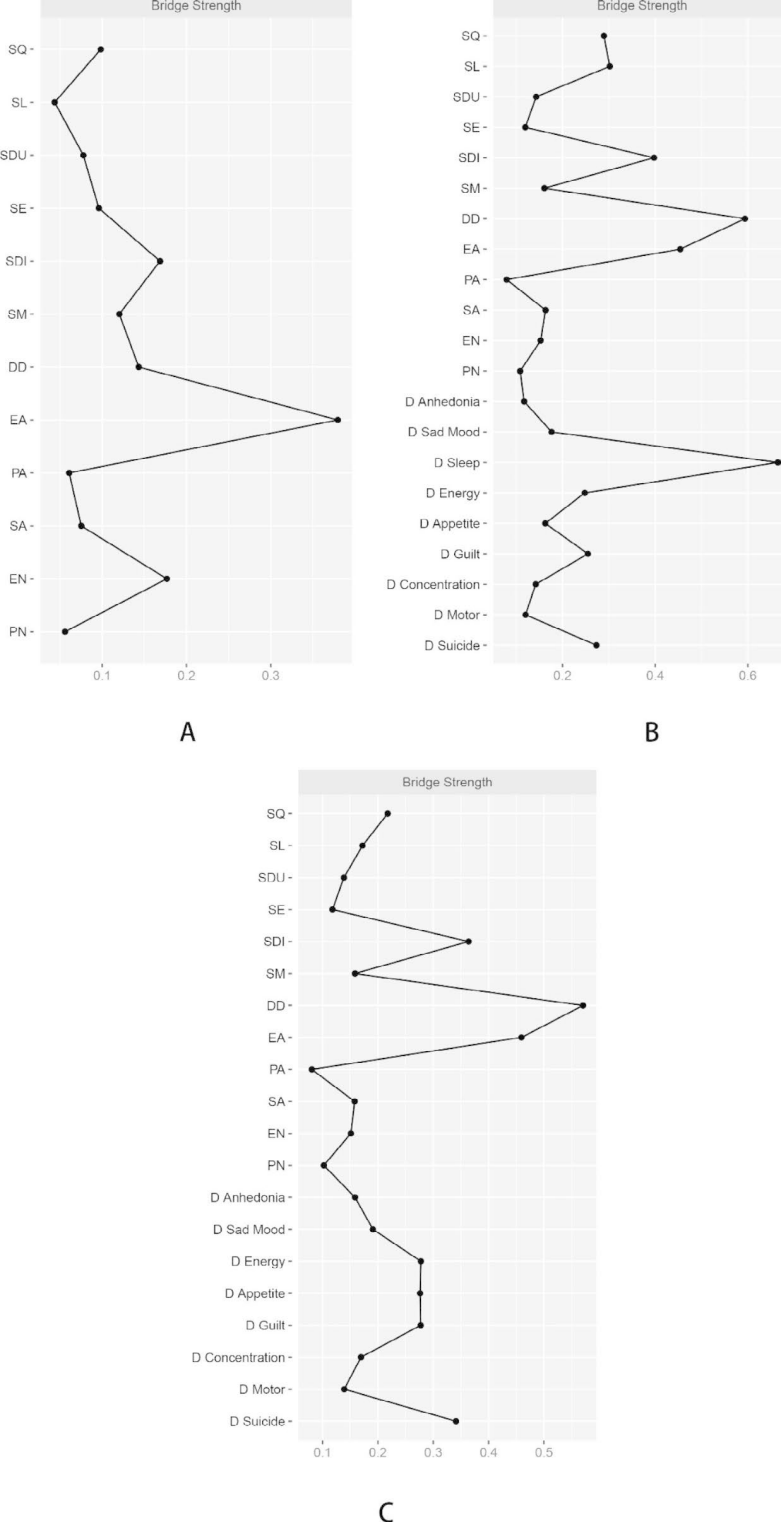
Bridge centrality indices of the sleep and abuse network, sleep, abuse and depression network (1), sleep, abuse and depression network (2). Note: Labels for sleep symptoms: SQ = sleep quality, SL = sleep latency, SDU = sleep duration, SE = sleep efficiency, SDI = sleep disturbance, SM = sleep medication, DD = daytime dysfunction. Labels for abuse: EA = emotional abuse, PA = physical abuse, SA = sexual abuse, EN = emotional neglect, PN = physical neglect. Labels for depressive symptoms: D Anhedonia = low interest or pleasure, D Sad Mood = feeling down and hopeless; D Sleep = sleeping difficulty, D Energy = lack of energy; D Appetite = poor appetite/overeating; D Guilt = guilt; D Concentration = difficulty in concentrating; D Motor = moving slowly/restlessness; D Suicide = suicidal thoughts. Strength is indicated by the thickness of lines between nodes, with thicker lines representing stronger ties

Two clusters of symptoms (CT and SD symptoms) were found to be bridged by several symptoms (Fig. 1A). The CT symptoms that are closest to SD symptoms were as follows (with SD symptoms in parentheses): emotional abuse (close to daytime dysfunction), and emotional neglect (close to sleep medication) (see Table S3 and Fig. 2A). The CT symptoms with the highest centrality were emotional abuse (betweenness = 1.64, closeness = 1.34, and strength = 1.03), physical abuse (betweenness = 1.35, closeness = 1.11) and sexual abuse (betweenness = 1.20, closeness = 0.60), whereas the SD symptoms with the highest centrality were sleep quality (closeness = 0.55; strength = 0.83), sleep disturbance (betweenness = 0.33, closeness = 0.83, and strength = 0.43), and daytime dysfunction (closeness = 0.99, strength = 0.26). According to the bridge strength, emotional abuse and sleep disturbance were the two most prominent bridge symptoms in this model (Fig. 3A).

### Model 2. Psychopathology of sleep disorder, childhood trauma and associated depression

Three clusters of symptoms (CT, SD and depressive symptoms) were found to be bridged by several symptoms (Fig. 1B). The depressive symptoms that are closest to CT symptoms and SD symptoms are as follows (with CT symptoms and SD symptoms in parentheses): sleeping difficulty (close to long sleep latency, poor sleep quality and sleep disturbance), suicidal thoughts (close to emotional abuse and use of sleep medication), and guilt (close to emotional abuse) (see Table S4 and Fig. 2B). The SD symptoms with the highest centrality were poor sleep quality (betweenness = 0.17, closeness = 0.42) and daytime dysfunction (closeness = 0.27, strength = 0.30), the CT symptoms with the highest centrality were emotional abuse (betweenness = 1.22, closeness = 0.36, and strength = 0.42) and physical abuse (betweenness = 1.31, closeness = 0.17), whereas the depressive symptoms with the highest centrality were suicidal thoughts (betweenness = 2.46, closeness = 1.69, and strength = 1.10), sleeping difficulty (betweenness = 1.22, closeness = 0.72, and strength = 1.69) and guilt (closeness = 0.98, strength = 1.10). Other symptoms with high centrality included feeling down and hopeless (closeness = 1.19) and lack of energy (strength = 1.52). According to the bridge strength, sleeping difficulty, daytime dysfunction, and emotional abuse were the three most prominent bridge symptoms in this model (Fig. 3B).

### Model 3. Psychopathology of sleep disorder, childhood trauma and associated depression (with the symptom of sleeping difficulty removed)

Three clusters of symptoms (CT, SD and depressive symptoms) were found to be bridged by several symptoms (Fig. 1C). The depressive symptoms that are closest to SD symptoms and CT symptoms are as follows (with CT symptoms and SD symptoms in parentheses): guilt (close to emotional abuse, poor sleep quality, long sleep latency and daytime dysfunction) and suicidal thoughts (close to emotional abuse and abnormal sleep duration) (see Table S5 and Fig. 2C). The SD symptoms with the highest centrality were sleep disturbance (betweenness = 0.73, closeness = 0.73) and daytime dysfunction (closeness = 0.41, strength = 0.43), the CT symptoms with the highest centrality were emotional abuse (betweenness = 1.62, closeness = 0.48, and strength = 0.52) and physical abuse (betweenness = 0.73, closeness = 0.12), whereas the depressive symptoms with the highest centrality were suicidal thoughts (betweenness = 2.62, closeness = 1.73, and strength = 0.87), feeling down and hopeless (betweenness = 0.63, closeness = 1.22, and strength = 0.75) and guilt (closeness = 1.14, strength = 1.22). Other symptoms with high centrality included sexual abuse (betweenness = 0.63) and lack of energy (strength = 1.65). According to the bridge strength, daytime dysfunction, emotional abuse, and sleep disturbance were the most prominent bridge symptoms in this model (Fig. 3C).

### Stability analyses

**Fig. 4:**
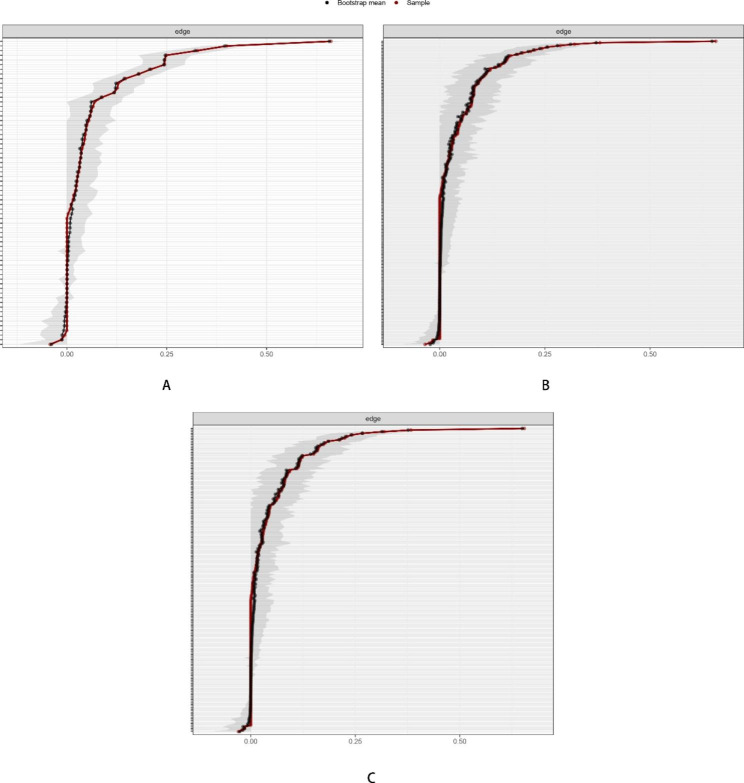
Accuracy of edge weight in the sleep and abuse network, the sleep, abuse and depression network (1) and the sleep, abuse and depression network (2)

The stability analyses showed that the network models were stable (see Fig. 4). Specifically, the edge weight stability analyses suggested that the tie strengths were reliably estimated. The node-dropping stability analyses suggested that the order of the nodes with regard to centrality was stable even after removing up to 50% of the nodes in each network. Strength centrality appeared to be the most stable among the centrality measures.

## Discussion

To the best of our knowledge, this was the first study to characterize the CT - SD symptom network of adolescents. We identified several core symptoms and bridge symptoms between CT and SD symptom clusters, as well as the bridge symptoms between CT and SD and depression symptom clusters. Overall, two common findings were yielded from in these networks. First, emotional abuse and sleep quality symptoms were core symptoms of CT and SD in all the networks, and with regard to the overlap between CT and SD, emotional abuse and sleep disturbance symptoms were found to be bridge symptoms that connected CT and SD. Second, with regard to the overlap between CT and SD and depressive symptoms, sleeping difficulty symptoms, daytime dysfunction symptoms, and emotional abuse were found to be bridge symptoms that connected CT and SD to depressive symptoms. However, with regard to the overlap between CT, SD and depressive symptoms with the symptom of sleeping difficulty removed, daytime dysfunction symptoms, emotional abuse, and sleep disturbance symptoms were found to be bridge symptoms that connected CT or SD to depressive symptoms.

Emotional abuse and poor sleep quality were identified as the central symptoms in the sleep and abuse network model. Some studies found that emotional abuse in childhood or adolescents could impact the sleep quality of the affected individuals into their adulthood [41–44], which might be explained by that emotional abuse occurring during an important developmental period might affect the stress regulatory mechanism [45]. However, these studies focused on the relationship between childhood emotional abuse and sleep problems of adults, different from our focus, which is the relationship between childhood emotional abuse and adolescent SD. Although the above findings could not directly support our view, they still indicate that emotional abuse and sleep quality symptoms are central to the sleep and abuse network, which is also an important finding of our study. These symptoms anchored the center of the network and had the strongest and most frequent relationships with other symptoms of CT and SD. Clinically, bridge symptoms can be viewed as transdiagnostic, indicating that targeted interventions may be effective for both disorders [46]. In a meta-analysis involving 28 cross-sectional and retrospective studies and a total of 84,164 subjects [47], Sandhya et al. investigated three possible reasons for the associations between CT and SD, which provided a basis for understanding bridge symptoms within CT-SD symptom network. According to this meta-analysis study, the most significant bridge symptoms between CT and SD included emotional abuse and sleep disturbance symptoms, which might be related to the increased corticotropin-releasing hormone (CRH) reactivity and increased brain activity; this also supported the association between CT and SD in adulthood [48–50]. In addition to alterations in the stress response system, adolescents who have experienced emotional abuse and physical abuse were more likely to have disrupted circadian rhythms, resulting in more sleep-related symptoms and even an increased risk of insomnia disorders [51, 52]. Besides, adolescents with emotional abuse may also be less likely to have optimal parental bonding/attachment styles [53], which has been shown to be associated with sleep problems dominated by sleep disturbance [54]. Given that the present study is the first study to characterize the CT-SD symptom network for adolescents, our findings might be helpful for further studies.

CT has been shown to be associated with depressive symptoms [55], and SD and depressive symptoms can also develop independently and affect each other [56]. Jessica et al. found an association between emotional neglect and SD in individuals with a history of depression [57], Luo et al. found that SD could partially mediate the relationship between different subtypes in CT and depression [58], and Li er al. found that SD was associated with a higher rate of suicidal ideation in adolescents with depression who had experienced CT except for sexual abuse [59]. Therefore, we inferred that depressive symptoms might affect the CT-SD correlation. To verify this view, we created a network model for the psychopathology of SD, CT and the associated depression and found that sleeping difficulty, daytime dysfunction, and emotional abuse might be bridge symptoms connecting CT or SD to depressive symptoms. However, due to the high risk of false positive result related to SD in PHQ-9, we rebuilt the model by removing the symptom of sleeping difficulty; the result showed that daytime dysfunction symptoms, emotional abuse, and sleep disturbance symptoms might bridge CT, SD and depressive symptoms. We can see from the above findings that emotional abuse and daytime dysfunction symptoms are always found to be bridge symptoms linking CT, SD and depressive symptoms in the two models, and after removing sleeping difficulty symptoms, daytime dysfunction symptoms became the most prominent bridge symptom linking CT, SD and depressive symptoms. The possible reason for this alteration might be the switching of the core symptom from sleeping difficulty symptoms to lack of energy in this modified network model. Ariel et al. found low energy and depressive symptoms were key predictors in a relative importance analysis on multiple domains of impairment, which were consistent with our findings [60]. Thus, depressive symptoms are highly likely to be a regulator for the CT-SD correlation.

Despite the strengths of the present study, there are also some limitations. First, due to the cross-sectional design, causal relationship and dynamic changes related to the association between CT, SD and depression could not be revealed [60]. Second, generalizability of our findings to other populations might be limited, and it is possible that data collected from individuals in a different age group could generate different network structures. Third, we did not mention the impact of gender on network analysis in this study, which may be due to the lack of sufficient data volume. In the future, we will expand the sample size even further to explore possible gender differences in greater detail.

In summary, we found that emotional abuse and poor sleep quality were core symptoms in the sleep and abuse network model, with emotional abuse and sleep disturbance being bridge symptoms. We also found that daytime dysfunction symptoms might be a link between CT, SD and depressive symptoms. Future studies are needed to test whether interventions targeting the core symptoms of emotional abuse could maximize the improvement of other symptoms as well as whether interventions targeting daytime dysfunction symptoms could disrupt the link between CT, SD and depressive symptoms. Hopefully, our findings could also inform the development of targeted interventions to minimize the impact of emotional abuse and daytime dysfunction symptoms.

## Conclusion

In conclusion, this network analysis revealed emotional abuse and poor sleep quality as the central symptoms in the CT-SD network structure among Chinese adolescent students, with daytime dysfunction as the bridge symptom in the CT-SD-depression network structure. Timely, multilevel interventions targeting the central symptoms and bridge symptoms may be effective in alleviating the co-occurrence of CT, SD and depression in this population.

## Electronic supplementary material

Below is the link to the electronic supplementary material.

Supplementary Material 1

## Data Availability

All data generated or analyzed during this study are included in this published article [and its supplementary information files].

## Data Availability

The raw data supporting the conclusions of this article will be made available by the authors, without undue reservation.

## Abbreviations

CT: childhood trauma
SD: sleep disorder
PSQI: The Pittsburgh sleep quality index
CTQ-SF: The Childhood Trauma Questionnaire-Short Form
PHQ-9: The Patient Health Questionnaire-9
ED: eating disorder
CDCS: China Depression Cohort Study
SQ: sleep quality
SL: sleep latency
SDU: sleep duration
SE: sleep efficiency
SDI: sleep disturbance
SM: sleep medication
DD: daily dysfunction
EA: emotional abuse
PA: physical abuse
SA: sexual abuse
EN: emotional neglect
PN: physical neglect
M/D: minimization/denial
GGMS: Gaussian graph models
CI: confidence interval
CS-coefficient: The centrality stability coefficient
