# Supplementary material for: The bridge symptoms of childhood trauma, sleep disorder and depressive symptoms: a network analysis
Source: Child Adolesc Psychiatry Ment Health. 2023 Jul 4;17:88. doi: 10.1186/s13034-023-00635-6 (PMC10320961; doi:10.1186/s13034-023-00635-6)
Supplement: Supplementary file 1 — Supplementary Material 1 [file 13034_2023_635_MOESM1_ESM.docx]

Table S1. Mean score for each dimension of the PSQI and CTQ-SF

|  | **Mean Score** |
| --- | --- |
| Sleep (PSQI, n=1188)^a^ |  |
| SQ: Sleep quality | 0.71 |
| SL: Sleep latency | 2.07 |
| SDU: Sleep duration | 0.29 |
| SE: Sleep efficiency | 0.63 |
| SDI: Sleep disturbance | 0.74 |
| SM: Sleep medication | 0.03 |
| DD: Daytime dysfunction | 0.62 |
| Abuse (CTQ-SF) |  |
| EA: Emotional abuse | 6.97 |
| PA: Physical abuse | 5.99 |
| SA: Sexual abuse | 5.60 |
| EN: Emotional neglect | 12.98 |
| PN: Physical neglect | 9.66 |

^a^113 respondents did not complete the PSQI during the survey.

Table S2. Mean score of each symptom in the PHQ-9

|  | **Mean score** |
| --- | --- |
| Depressive symptoms (PHQ-9) |  |
| PHQ-1: Low interest or pleasure | 0.41 |
| PHQ-2: Feeling down, hopeless | 0.30 |
| PHQ-3: Trouble sleeping | 0.37 |
| PHQ-4: Tired or little energy | 0.31 |
| PHQ-5: Poor appetite/overeating | 0.34 |
| PHQ-6: Guilt | 0.39 |
| PHQ-7: Trouble concentrating | 0.26 |
| PHQ-8: Moving slowly/restless | 0.19 |
| PHQ-9: Suicidal thoughts | 0.21 |

Table S3. Strength of edges in the sleep and abuse network

| Edge | Partial correlation coefficient |
| --- | --- |
| DD-EA | 0.12 |
| SM-SA | 0.07 |
| SQ-EA | 0.06 |
| PA-PN | 0.05 |
| SE-PN | 0.05 |
| SDU-EN | 0.04 |
| SM-EA | 0.04 |
| SE-EN | 0.03 |
| DD-PA | 0.03 |
| SQ-EN | 0.03 |
| SL-PA | 0.03 |
| SL-SE | 0.03 |
| SDU-EA | 0.03 |

Note: Edges were included only if the absolute value of the edge was equal to or greater than the cutoff score calculated for the glasso network (0.03). The cutoff score represents a strength greater than that of 75% of the edges. Labels for sleep symptoms: SQ=sleep quality, SL=sleep latency, SDU=sleep duration, SE=sleep efficiency, SDI=sleep disturbance, SM=sleep medication, DD=daytime dysfunction. Labels for abuse: EA=emotional abuse, PA=physical abuse, SA=sexual abuse, EN=emotional neglect, PN=physical neglect.

Table S4. Strength of edges in the sleep, abuse and depression network (1)

| Edge | Partial correlation coefficient |  |
| --- | --- | --- |
| SDU-SE | 0.32 |  |
| SL-D.Sleep | 0.29 |  |
| DD-D.Energy | 0.22 |  |
| SQ-D.Sleep | 0.16 |  |
| SDI-D.Appetite | 0.10 |  |
| EA-D.Suicide | 0.09 |  |
| SDI-D.Sleep | 0.09 |  |
| SDI-EA | 0.08 |  |
| EA-D.Guilt | 0.08 |  |
| SDI-D.Sad.Mood | 0.08 |  |
| SM-D.Suicide | 0.07 |  |
| EA-D.Sad.Mood | 0.07 |  |
| SM-SA | 0.06 |  |
| DD-D.Sleep | 0.06 |  |
| DD-D.Appetite | 0.06 |  |
| PA-D.Sleep | 0.05 |  |
| DD-D.Guilt | 0.05 |  |
| SA-D.Motor | 0.05 |  |
| EN-D.Suicide | 0.05 |  |
| SQ-D.Guilt | 0.05 |  |
| SE-PN | 0.04 |  |
| DD-D.Concentration | 0.04 |  |
| SDU-D.Suicide | 0.04 |  |
| DD-EA | 0.04 |  |
| SDU-EN | 0.04 |  |
| EA-D.Anhedonia | 0.03 |  |
| SDU-D.Guilt | 0.03 |  |
| SE-EN | 0.03 |  |
| SQ-D.Concentration | 0.03 |  |
| SE-D.Motor | 0.03 |  |

Note: Edges were included only if the absolute value of the edge was equal to or greater than the cutoff score calculated for the glasso network (0.03). The cutoff score represents a strength greater than that of 75% of the edges. Labels for sleep symptoms: SQ=sleep quality, SL=sleep latency, SDU=sleep duration, SE=sleep efficiency, SDI=sleep disturbance, SM=sleep medication, DD=daytime dysfunction. Labels for abuse: EA=emotional abuse, PA=physical abuse, SA=sexual abuse, EN=emotional neglect, PN=physical neglect. Labels for depressive symptoms: D Anhedonia=low interest or pleasure, D Sad Mood=feeling down and hopeless, D Sleep=sleeping difficulty, D Energy=lack of energy, D Appetite=poor appetite/overeating, D Guilt=guilt, D Concentration=difficulty in concentrating, D Motor=moving slowly/restlessness; D Suicide=suicidal thoughts.

Table S5. Strength of edges in the sleep, abuse and depression network (2)

| Edge | Partial correlation coefficient |
| --- | --- |
| SDI-D.Appetite | 0.11 |
| EA-D.Suicide | 0.09 |
| SDI-EA | 0.08 |
| SDI-D.Sad.Mood | 0.08 |
| EA-D.Guilt | 0.08 |
| SM-D.Suicide | 0.07 |
| DD-D.Appetite | 0.07 |
| SL-D.Appetite | 0.07 |
| EA-D.Sad.Mood | 0.07 |
| SL-D.Suicide | 0.07 |
| SM-SA | 0.06 |
| SQ-D.Guilt | 0.06 |
| DD-D.Guilt | 0.06 |
| SA-D.Motor | 0.05 |
| EN-D.Suicide | 0.05 |
| SE-PN | 0.04 |
| DD-D.Concentration | 0.04 |
| SDU-D.Suicide | 0.04 |
| DD-EA | 0.04 |
| SQ-D.Concentration | 0.04 |
| SDU-EN | 0.04 |
| EA-D.Anhedonia | 0.04 |
| SDI-D.Energy | 0.04 |
| SDU-D.Guilt | 0.03 |
| SE-EN | 0.03 |
| SQ-D.Anhedonia | 0.03 |
| SE-D.Motor | 0.03 |
| SDU-DD | 0.03 |
| PA-D.Motor | 0.03 |
| SDI-D.Guilt | 0.03 |
| SQ-EA | 0.03 |

Note: Edges were included only if the absolute value of the edge was equal to or greater than the cutoff score calculated for the glasso network (0.03). The cutoff score represents a strength greater than that of 75% of the edges. Labels for sleep symptoms: SQ=sleep quality, SL=sleep latency, SDU=sleep duration, SE=sleep efficiency, SDI=sleep disturbance, SM=sleep medication, DD=daytime dysfunction. Labels for abuse: EA=emotional abuse, PA=physical abuse, SA=sexual abuse, EN=emotional neglect, PN=physical neglect. Labels for depressive symptoms: D Anhedonia=low interest or pleasure, D Sad Mood=feeling down and hopeless, D Sleep=sleeping difficulty, D Energy=lack of energy, D Appetite=poor appetite/overeating, D Guilt=guilt, D Concentration=difficulty in concentrating, D Motor=moving slowly/restlessness; D Suicide=suicidal thoughts.
